# Sub-optimal CD4 reconstitution despite viral suppression in an urban cohort on Antiretroviral Therapy (ART) in sub-Saharan Africa: Frequency and clinical significance

**DOI:** 10.1186/1742-6405-5-23

**Published:** 2008-10-28

**Authors:** Damalie Nakanjako, Agnes Kiragga, Fowzia Ibrahim, Barbara Castelnuovo, Moses R Kamya, Philippa J Easterbrook

**Affiliations:** 1 Infectious Diseases Institute, Facluty of Medicine, Makerere University Kampala, Uganda; 2 Department of HIV/GUM, King's College London, SWZ, London, UK

## Abstract

**Background:**

A proportion of individuals who start antiretroviral therapy (ART) fail to achieve adequate CD4 cell reconstitution despite sustained viral suppression. We determined the frequency and clinical significance of suboptimal CD4 reconstitution despite viral suppression (SO-CD4) in an urban HIV research cohort in Kampala, Uganda

**Methods:**

We analyzed data from a prospective research cohort of 559 patients initiating ART between 04/04–04/05. We described the patterns of SO-CD4 both in terms of:- I) magnitude of CD4 cell increase (a CD4 count increase < 50 CD4 cells/μl at 6 months, <100 cells/μl at 12 months; and <200 cells/μl at 24 months of ART) and II) failure to achieve a CD4 cell count above 200 cells/μl at 6,12 and 24 months of ART. Using criteria I) we used logistic regression to determine the predictors of SO-CD4. We compared the cumulative risk of clinical events (death and/or recurrent or new AIDS-defining illnesses) among patients with and without SO-CD4.

**Results:**

Of 559 patients initiating ART, 386 (69%) were female. Median (IQR) age and baseline CD4 counts were 38 yrs (33–44) and 98 cells/μl (21–163) respectively; 414 (74%) started a d4T-based regimen (D4T+3TC+NVP) and 145 (26%) a ZDV-based regimen (ZDV+3TC+EFV). After 6, 12 and 24 months of ART, 380 (68%), 339 (61%) and 309 (55%) had attained and sustained HIV-RNA viral suppression. Of these, 78 (21%), 151 (45%) and 166 (54%) respectively had SO-CD4 based on criteria I), and 165(43%), 143(42%) and 58(19%) respectively based on criteria II). With both criteria combined, 56 (15%) and 129 (38%) had SO-CD4 at 6 and 12 months respectively. A high proportion (82% and 58%) of those that had SO-CD4 at 6 months (using criteria I) maintained SO-CD4 at 12 and 24 months respectively. There were no statistically significant differences in the incidence of clinical events among patients with [19/100PYO (12–29)] and without SO-CD4 [23/100PYO (19–28)].

**Conclusion:**

Using criteria I), the frequency of SO-CD4 was 21% at 6 months. Majority of patients with SO-CD4 at 6 months maintained SO-CD4 up to 2 years. We recommend studies of CD4 T-cell functional recovery among patients with SO-CD4.

## Introduction

There is considerable variability in the magnitude and rate of CD4 T cell count recovery in Human immunodeficiency virus type 1 (HIV-1)-infected individuals, receiving antiretroviral therapy (ART). Most patients show a progressive rise in CD4 T cell counts after initiation of ART [1,2], however, some patients fail to attain CD4 counts that exceed 200 cells/μl, and thus remain profoundly immune suppressed despite suppression of HIV-1 viral replication. The frequency of suboptimal immunological response to ART despite viral suppression varies between 7–20% [3-7] depending on the duration of ART and definition of SO-CD4 (see Table 1). There is limited data on the frequency of suboptimal CD4 reconstitution despite viral suppression (SO-CD4) in sub-Saharan Africa (SSA) where most patients initiate ART at advanced stages of HIV/AIDS [1,8,9] amidst a high background risk acute infections. Moreover, there are conflicting reports about the correlation of SO-CD4 with clinical morbidity and susceptibility to opportunistic infections [4,5,10]. We hypothesize that patients with SO-CD4 are at increased risk of clinical events (death and/or recurrent or new AIDS-defining illnesses). In this study, we determined the frequency, predictors and clinical significance of SO-CD4 reconstitution as evidenced by of occurrence of acquired immunodeficiency syndrome (AIDS)-related clinical events (recurrent or new opportunistic infection and/or death).

**Table 1:** Published definitions of suboptimal CD4 reconstitution among patients with viral suppression

**Author**	**Cohort description**	**Duration of follow up**	**Baseline CD4 count** **Median (IQR) cells/μl**	**ART regimen**	**Definition of SO-CD4** **and Frequency**	**Clinical events among suboptimal responders versus complete responders**
Lawn [5]	596 ART-naïve patients at a community HIV clinic in Cape Town, South Africa	48 weeks	97 (50–153)	2 NRTIs + 1 NNRTI	Increase < 50 cells/μl at 12 months SO-CD4-7%	No data
Tuboi [6]	1914 ART naïve in HIV clinics in Africa, Latin America and Asia (ART-LINC)	6 months	137 (49–240)	2 NRTIs +1 NNRTI (57.3%) 2 NRTIs + PI (29%)	Increase < 50 cells/μl at 6 months SO-CD4-19%	No data
Tan [7]	Prospective observational cohort of 404 ART naïve patients in an HIV clinic at the University of Alabama, Birmingham, US	9 months	Mean = 214(SD 260)	2 NRTIs +1 NNRTI (49%) 2 NRTIs + PI (40%)	Increase < 50 cells/μl at 6 months SO-CD4-8.7%	Patients with discordant CD4 and virologic responses were 2.28 times more likely to develop opportunistic infections/death aOR 2.28(1.31–4.00)
Teixeira [10]	21 ART naïve patients attending an Immunology clinic at 2 sites in the US (Ohio and San Francisco)	12 months	170 (90–276)	No data	Increase < 100 cells/μl at 1 year SO-CD4-57%	No data
Jevtovic [18]	Retrospective study of 446 patients at an HIV center in the Institute for Tropical diseases, Belgrade 52% ART naïve	33 months	Mean 115 ± 95	2 NRTIs + PI (34%) 2 NRTIs+1 NNRTI (40%)	Absolute CD4 count of < 400 cells/μl at 2–3 years SO-CD4-39%	Clinical events were no higher among virologic only responders than complete CD4 & virologic responders
Florence [12]	EuroSida study – Prospective cohort of 8500 ART naïve patients in 63 hospitals of 20 European countries;	12 months	150 (80–228);	2 NRTIs + PI (86%) 2NRTIs +NNRTI (10.8%)	Increase < 50 cells/μl at 6 months SO-CD4-29%	No data
Piketty [3]	Prospective cohort of I62 ART experienced but PI -naive patients at an HIV clinic in France	12 months	Mean 69 ± 5.0	2 NRTIs + PI	Increase < 50 cells at 12 months SO-CD4-10.5%	Higher Incidence of AIDS-defining events among virologic only responders (4/7) than complete responders (7/92) [P = 0.07]
Grabar [4]	Prospective cohort of 2236 PI naïve patients from 68 hospitals in France	18 months	150(65–263)	2NRTIs +PI	Increase < 50 cells/μl at 6 months SO-CD4-17.3%	Patients with only good virologic responses were 3 times more likely to develop an AIDS-defining illness/death than complete responders RR 3.38 (2.28–5.02)
Kaufmann [13]	Swiss cohort study – 293 ART naïve patients	5 years	180 (60–311)	2NRTIs +PI (98%)	Absolute CD4 count below 500 cells/μl at 5 yrs SO-CD4-35.8%	Higher incidence of CD4 category B events among incomplete responders (13.3%) than incomplete responders (9.6%) p > 0.05

## Patients and methods

### Study site

The Infectious Diseases Institute (IDI) is a private-public partnership institution that is a center of excellence in HIV care, training, and research at Makerere University Medical School and Mulago Teaching Hospital in Kampala, Uganda. Since 2004, the IDI clinic (IDC) has provided free care to HIV positive patients, and by December, 2007, IDC had enrolled 20,000 patients into HIV care, of whom 13,000 are in active follow-up and 4700 have initiated ART according to WHO and Uganda Ministry of Health guidelines. The clinic has 15 exam rooms and is staffed by 20 physicians, 30 nurses, and 10 counselors and patients are reviewed monthly. The drugs are provided by the Global Fund (a generic combined formulation of stavudine [d4T, lamivudine [3TC], and nevirapine [NVP] or by the US President's Emergency Plan for AIDS Relief (a combined formulation of zidovudine [ZDV] and 3TC plus efavirenz [EFZ]/nevirapine [NVP]. Our research was approved by the Uganda National Council of Science and Technology.

### Study subjects, procedures and measurements

From April 2004 to April 2005, 559 consecutive HIV-infected patients initiating ART were enrolled into a prospective observational research cohort if they attended the clinic regularly (having attended at least 2 clinic visits in the 6 months prior to ART initiation). Daily co-trimoxazole prophylaxis was provided and patients allergic to co-trimoxazole were given dapsone. Adherence to ART was encouraged by at least 3 individual and group counseling sessions. Patients are reviewed monthly by the general clinic physicians that evaluated among others; adherence to medication, toxicities and acute infections. Patients are evaluated by the study physicians every 3 months or earlier if they develop any illness. HIV RNA viral loads, complete blood counts and CD4 lymphocyte counts are tested at 6 monthly intervals.

### Definitions of suboptimal CD4 reconstitution despite sustained viral suppression (SO-CD4)

In this study, we used (i) previously used definitions of SO-CD4 in terms of the magnitude of the CD4 cell increase [a CD4 count increase of < 50 CD4 cells/μl after 6 months of ART [3-6]; <100 cells/μl increase after 12 months [11]; and <200 cells/μl after 24 months; and (ii) failure to achieve a CD4 cell count above a threshold of 200 cells/μl at 6, 12 and 24 months; the critical CD4 count below which patients remain highly susceptible to opportunistic infections.

### Statistical analysis

Patients were included in the analysis if they had attained and sustained HIV-RNA viral load ≤ 400 copies per ml at 6, 12 and 24 months. The chi square test was used to compare the baseline clinical characteristics of patients with and without SO-CD4 and the level of significance was 0.05. Proportions of patients with SO-CD4 were calculated using the two criteria independently and with the two criteria combined. The combination of the two criteria was the intersection of patients with SO-CD4 on both criteria I) and II). Logistic regression by stepwise model selection was used to analyze predictors of SO-CD4. The independent variables included age, sex, baseline CD4 cell counts, body mass index (BMI), baseline hemoglobin, initial ART regimen, magnitude of CD4 increase in first 6 months and Hepatitis B surface Antigen sero-status. Variables were included in the multivariate model if they had a p value ≤ 0.25 on bivariate analysis. The proportions of clinical events were examined among patients with and without SO-CD4. In addition, the cumulative risk of development of AIDS-related clinical events was estimated by Kaplan-Meier analysis. Patients were censored on the occurrence of an AIDS-related clinical event (the primary outcome) as required by the survival analysis technique. Differences between the survival curves were tested using the log-rank test.

## Results

### Baseline characteristics

Of 559 patients initiating ART, 386 (69%) were female, with a median age of 38 yrs (IQR 33–44), and a median CD4 count of 98 cells/μl (IQR 21–163). Half 283(51%) of the patients had severe immune suppression with CD4 counts below 100 cells/μl at initiation of ART. Majority of patients, 414 (74%) started a d4T-based regimen (D4T+3TC+NVP) and 145 (26%) a ZDV-based regimen (ZDV+3TC+EFV). Baseline characteristics were comparable among optimal and sub-optimal responders apart from the baseline CD4 count that was significantly higher among sub-optimal than optimal responders. Patients with a lower BMI at initiation of therapy were more likely to have SO-CD4 after 12 months although the difference was no longer significant after 24 months of ART. Similarly, patients that initiated a ZDV-based regimen were more likely to have SO-CD4 at 6 and 12 months although the difference was no longer significant after 24 months of ART (see Table 2). At 6 months, 93 (17%) were excluded from analysis because; 6 did not have laboratory tests, 19 were lost to follow up and 68 were dead. The patients that were lost to follow up had a median baseline CD4 count of 144(11–189) cells/μl although their CD4 reconstitution could not be classified since they could not be accessed for a second measurement. The majority (64%) of the deaths among patients with viral suppression were not HIV-related and the causes of death included among others; drug-induced hepato-toxicity, lactic acidosis, road traffic accidents and obstetric deaths (data not shown). The median follow up was 22(IQR 3–22) months.

**Table 2:** Baseline characteristics of patients with sustained viral suppression over 24 months in the Infectious Diseases. Institute research cohort

**Duration of HAART**	6 months **(N = 380)**			12 months **(N = 339)**			24 months **(N = 309)**		
**Variable**	**CD4 increase < 50 cells/μl**	**CD4 increase ≥ 50 cells/μl**	P value*	**CD4 increase < 100 cells/μl**	**CD4 increase > 100 cells/μl**	**P value**	**CD4 increase < 200 cells/μl**	**CD4 increase > 200 cells/μl**	**P value**
**SO-CD4**	78 (21%)	302 (79%)		151 (45%)	188 (55%)		166 (54%)	143 (46%)	

**Age **(yrs), [median (IQR)]	38(33–44)	37(33–43)		38(33–45)	37(32–44)		37(32–44)	41(34–45)	
**≤ 35**	30(38%)	126(42%)	0.60	53(35%)	84(45%)	0.14	58(35%)	69(48%)	0.02
**Gender**									
Female	53(68%)	216(71%)	0.54	108(71%)	136(72%)	0.87	120(72%)	107(74%)	0.62
**BMI increase **[median (IQR)]	0.83 (-0.4–2.0)	1.31 (0–2.56)	0.49	1.4(0.0–2.5)	2.3(0.7–3.9)	<0.01	1.3(0–2.5)	2.8(0.7–4.6)	0.86
									
**HAART regimen initiated**									
D4T-3TC-EFZ/NVP	40(14%)	242(86%)		94(62%)	154(82%)		117(38%)	110(36%)	0.62
AZT-3TC-EFZ/NVP	38(39%)	60(61%)	<0.01	57(38%)	34(18%)	<0.01	49(16%)	33(11%)	
									
**Baseline CD4 count **[Median(IQR)]	123(84–186)	99(29–162)	<0.01	122(78–189)	96(14–162)	<0.01	119(77–176)	87(11–158)	<0.01
									
Hepatitis BSAg * (270 tests done)									
**Positive**	8(31%)	18(69%)	0.17	10(7%)	11(6%)	0.93	10(6%)	11(8%)	0.24
**Negative**	59(20%)	233(80%)		114(75%)	145(77%)		131(79%)	102(71%)	

* NOTE: The analysis was limited to only 270 patients that were tested for Hepatitis B surface antigen sero-status

### Suboptimal CD4 reconstitution

**Figure 1:**
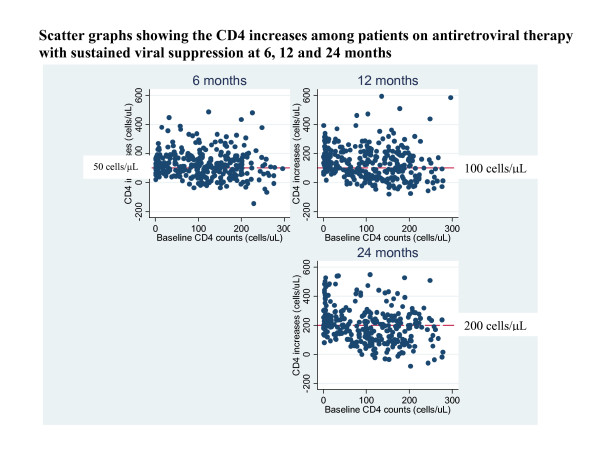
Scatter graphs showing the CD4 increases among patients on antiretroviral therapy with sustained viral suppression at 6, 12 and 24 months.

After 6, 12 and 24 months of ART, 380 (68%), 339 (61%) and 309 (55%) had attained and sustained HIV-RNA viral suppression. Of these, 78 (21%), 151 (45%) and 166 (54%) respectively had SO-CD4 using the CD4 increase criteria (described in the methods section). Of the patients with SO-CD4 at 6 months, 64/78 (82%) and 45/78 (58%) still had SO-CD4 after 12 months and 24 months respectively. By the end of 2 years on ART the overall median change in CD4 cell count and percentage was 193(104–273) and 11.5% (IQR 8.6–14.6) respectively though it was 77 [IQR 25–127] cells/μl and 7.2% [IQR 4.1–9.6] respectively among patients with SO-CD4 (see figure 1).

Using the CD4 threshold criterion, 165/380 (43%), 143/339(42%) and 58/309 (19%) had SO-CD4 at 6, 12 and 24 months of ART respectively. Of the patients with SO-CD4 at 6 months, 112/165 (68%) and 46/165 (41%) still had SO-CD4 at 12 and 24 months respectively. By 2 years on ART the median change in CD4 cell count and percentage was 54 [IQR 22–99] cells/μl and 6.4% [IQR 3.5–8.1] among patients with SO-CD4 based on threshold definition (see table 3).

**Table 3:** Suboptimal CD4 reconstitution and clinical events among patients with sustained viral suppression in the infectious.

**Duration of HAART**	**0–6 months (N = 380)**			**6–12 months (N = 339)**			**12–24 months (N = 309)**		
**Variable**	**Suboptimal response**	**Optimal response**	**P value**	**Suboptimal response**	**Optimal response**	**P value**	**Suboptimal response**	**Optimal response**	**P value**

**i)SO-CD4 magnitude definition**	**78(21%)**	**302(79%)**		**151(45%)**	**188(55%)**		**166(54%)**	**143(46%)**	
**OI events**	11(14%)	77(25%)	0.04	14(9%)	9(5%)	0.13	8(5%)	4(3%)	0.40
									
**ii)Threshold definition**	**165(43%)**	**215(57%)**		**143(42%)**	**196(58%)**		**58(18%)**	**309(82%)**	
**OI events**	49(30%)	66(31%)	0.90	11(8%)	12(6%)	0.66	1(0%)	11(8%)	0.70
									
**iii) Definitions i & ii combined**	**56(15%)**	**324(85%)**		**129(38%)**	**213(62%)**		**3(1%)**	**306(99%)**	
**OI events**	8(10%)	80(26%)	0.12	9(6%)	14(7%)	0.36	2(1%)	11(8%)	0.70

Diseases Institute research cohort: we defined suboptimal CD4 reconstitution as i) CD4 count increase of either of a) less than 50 CD4 cells/μl at 6 months [6] b) <100 cells/μl in the first year of therapy [10] and c) <200 cells/μl after 2 years of HAART; ii) CD4 T cell threshold of <200 cells/μl.

With both criteria combined, 56/380 (15%), 129/339 (38%) and 3/309 (1%) had SO-CD4 after 6, 12 and 24 months of ART respectively. Of the patients with SO-CD4 at 6 months, 42/56 (75%) still had SO-CD4 after 12 months of therapy.

### Predictors of SO-CD4

Patients with baseline CD4 counts of 50–199 cells/μl were more likely to have SO-CD4 than those with baseline CD4 counts of 0–49 cells/μl at 6 months [OR 2.5(1.1–5.5) P = 0.03] and at 12 months [OR 2.9(1.6–5.4) P = 0.001]. In addition, patients who initiated zidovudine-containing ART regimen were more likely to have SO-CD4 than patients on stavudine-containing ART at 6 months [OR 4.5(2.4–8.3) P < 0.001] and at 12 months [3.6(2.0–6.4) P < 0.001]. Other factors like age, sex, body mass index and hemoglobin level were not significant predictors of SO-CD4.

### Clinical significance of suboptimal CD4 reconstitution

**Figure 2:**
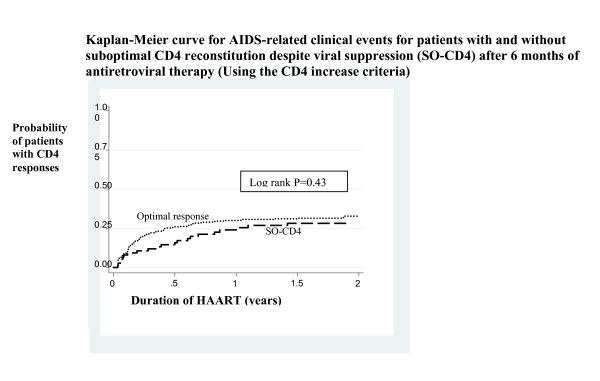
Kaplan-Meier curve for AIDS-related clinical events for patients with and without suboptimal CD4 reconstitution despite viral suppression (SO-CD4) at 6 months of antiretroviral therapy (Using the CD4 increase criteria).

Overall, there were 22 clinical events/100 PYO (18–26) among patients with sustained viral suppression. There were no statistically significant differences in the clinical events among patients with [19/100PYO (12–29)] and without SO-CD4 (using the CD4 increase criteria) [23/100PYO (19–28) p = 0.43] see Figure 2. The commonest opportunistic infections (OIs) were oral candidiasis (31%), bacterial pneumonia (22%), and tuberculosis (16%). Apart from oral candidiasis that occurred only at CD4 counts below 100 cells/μl, there were no significant differences in CD4 counts depending on the specific OIs. Despite viral suppression, 14% and 30% of the OIs in the first 6 months of therapy occurred among patients with SO-CD4 using the CD4 increase and threshold criteria respectively (see Table 3).

## Discussion

In this population with good rates of viral suppression as was previously reported [12], the frequency of SO-CD4, using the CD4 increase criteria, was 21%, 45% and 54% at 6,12 and 24 months respectively. Our findings are comparable to results from other developing countries (Africa, Latin America and Asia) where 19% of patients had SO-CD4 using a similar criteria of a CD4 increase of < 50 cells/μl after 6 months of ART [6]. Similarly, the frequency of SO-CD4 at 6 and 12 months is comparable to what has been reported in industrialized countries that used similar criteria [4,11,13]. Overall, our results show similar profiles of CD4 reconstitution in both the developing and industrialized countries despite the challenges with infrastructure for care delivery in sub-Saharan Africa.

We found that patients with baseline CD4 counts of 50–199 cells/μl were about 3 times more likely to have SO-CD4 than those with baseline CD4 counts of 0–49 cells/μl. Our results are similar to reports from South Africa where patients in the lower CD4 stratum had a higher gradient of CD4 increase [5]. This is contrary to previous reports that advanced pre-treatment immunodeficiency is associated with diminished capacity to restore quantitative and functional CD4 T cell responses during antiretroviral therapy [14,15]. We attribute our results to the peripheral expansion and/or redistribution of CD4 T cells that is described in the initial phase of CD4 reconstitution on ART [16]. Our results imply that the CD4 increase criteria of SO-CD4 is not enough in a setting where patients present to hospitals and HIV care units with untreated advanced HIV disease [1,17]. In addition, we used a threshold of 200 cells/μl below which patients were classified as SO-CD4 since this gives an indication of the general susceptibility to opportunistic infections. Using the CD4 threshold criteria, we found that 43%, 42% and 19% had SO-CD4 at 6, 12 and 24 months respectively; thereby remaining at risk of opportunistic infections.

Patients that initiated therapy with a zidovudine-containing regimen were 3.6 times more likely to develop SO-CD4 than patients on a d4T-containing regimen and we attribute this to the myelosuppressive effects of zidovudine [18]. We interpret these results cautiously because only 26% of our patients initiated a zidovudine-containing regimen and they were not randomized. However, evidence in the US shows that use of a protease inhibitor (PI)-based regimen is protective against poor immune reconstitution [6,19] because PIs modulate activation of peripheral blood CD4 T cells and decrease their susceptibility to apoptosis [20]. Since the long term prognosis of patients exhibiting discordant responses remains unknown [3], we need to explore the use of the newer and less toxic first line regimens [21] for patients at risk of SO-CD4.

Age was not a significant predictor of SO-CD 4 in our study and this is consistent with what was reported in a US cohort [22]. However, some previous studies showed that age above 30 years was associated with SO-CD4 [5,11] because it correlated with thymic involution yet preserved thymic function is necessary for adequate CD4 T cell recovery [11,23]. Similarly, hepatitis B co-infection did not predict SO-CD4 as was recently reported that hepatitis B co-infection had no impact on the response to ART regarding viral suppression and immune recovery[24].

Majority of the patients with SO-CD4 after 6 months, using either of the criteria, still had SO-CD4 at 12 months despite sustained HIV-RNA viral suppression. Since patients with SO-CD4 at 6 months are likely to maintain the phenomenon, they may need evaluation of the recovery of CD4 cell function, more so in Africa where there is an increased background risk of opportunistic infections. It is possible that the CD4 cells do not recover both in absolute numbers and function because of the high levels of T-cell activation in Africans due to frequent infections by the various pathogens endemic in the region [10,25,26].

It is also likely that these patients may require extended periods of prophylaxis against opportunistic infections. Our analysis was however limited to recovery of peripheral CD4 T cell counts and not CD4 T cell function. We recommend studies to examine other markers of recovery of immunological function among patients with SO-CD4.

We found that about a third of the opportunistic infections occurred among patients with SO-CD4 reconstitution as defined by either the CD4 increase or the threshold criteria. Similar to what has been reported in other cohorts, most of the AIDS-related events occurred in the first 6 months [27-29] and the spectrum of opportunistic infections was similar to what was found among patients at Mulago hospital where most patients with advanced HIV disease were hospitalized with severe bacterial pneumonias and tuberculosis [17]. More AIDS-related events were recorded among patients without SO-CD4 and we postulate that immune reconstitution inflammatory syndrome (IRIS) contributed to this difference [9]. However, Kaplan-Meier analysis showed no statistically significant differences in the rates of AIDS-related clinical events among patients with and without SO-CD4 in the setting of HIV-RNA viral suppression. On the contrary, in industrialized countries, patients with SO-CD4 (using similar criteria) have previously been reported to have a higher risk of developing an AIDS-related clinical events [4,30]. In the Swiss cohort, suboptimal responders had a 1.5 fold higher incidence of opportunistic infections than the complete CD4 responders [14]. However, we are cautious to compare our results with the latter cohort because the authors used a CD4 threshold below 500 cells/μl after 5 years of ART to define SO-CD4 at a frequency of 35.8%. We need to consider SO-CD4 after longer periods of follow up like has been done in the industrialized countries. Our results add to the emphasis that viral load testing is required for monitoring patients on ART in resource limited settings [31] especially those patients that present with unsatisfactory CD4 reconstitution in order to guide treatment decisions for this subgroup of patients.

The findings in this study are strengthened by the relatively homogenous study population of ART-naive individuals receiving ART at a single facility using standardized clinical protocols. Our patients used NNRTI-based ART regimen that are used in most HIV care facilities in Africa so our results can be generalized to most patients in Africa however they are limited to patients with sustained HIV-RNA viral suppression which, among others, is the ultimate goal of ART. We need to design studies of interventions for patients on ART with poor immune reconstitution and minimize the time spent with CD4 counts below the 200 cells/μl critical threshold. It is important to note that adherence to ART and previous exposure to ART were not considered to contribute to SO-CD4 in our study since all patients were naïve to ART and patients were included in the analysis only if they had HIV-RNA viral load < 400 copies/ml which we used as a proxy for good adherence.

## Conclusion

The frequency of SO-CD4 is high in SSA and many of the patients with SO-CD4 at 6 months maintain the phenomenon up to 2 years of therapy. However, the rates of AIDS-related clinical events were no higher in those with SO-CD4. We recommend studies of CD4 T-cell functional recovery among patients with SO-CD4.

## Competing interests

The authors declare that they have no competing interests.

## Authors' contributions

DN conceived of the study, and participated in the design, data analysis, interpretation of data, drafting and revising the paper. AK participated in the study design and statistical analysis. FI participated in the statistical analysis. BC participated in the acquisition of data, coordination of the study and in revising the paper. MRK made substantial contribution to the conception, design and coordination of the study. PJE made substantial contribution study design, statistical analysis and revision of the paper. All authors read and approved the final manuscript.
